# Biomimetic Nanoplatform for Fibrosis Remodeling and Anti‐PD‐1 Potentiation via Cu^+^/Ca^2+^ Ion Dysregulation and TGF‐β Inhibition in PDAC

**DOI:** 10.1002/advs.78436

**Published:** 2026-10-22

**Authors:** Jianmei Li, Qinlong Wen, Jie Dai, Li Yang, Feng Xiong, Zhigong Wei, Xingchen Peng

**Affiliations:** ^1^ Department of Biotherapy Cancer Center West China Hospital Sichuan University Chengdu Sichuan People‘s Republic of China; ^2^ Nuclear Physics and Medical Research Key Laboratory of Sichuan Province Sichuan University Chengdu Sichuan People‘s Republic of China; ^3^ Department of High Altitude Medicine Center for High Altitude Medicine West China Hospital Sichuan University Chengdu Sichuan People‘s Republic of China

**Keywords:** fibrotic barrier disruption, ion interference therapy, pancreatic carcinoma, targeted delivery nanoplatform

## Abstract

Pancreatic ductal adenocarcinoma (PDAC) remains refractory to immunotherapy, largely due to the dense fibrotic stroma that promotes immune evasion and restricts drug penetration. Here, we report a multifunctional biomimetic nanoplatform (RPm@CC‐LY) that simultaneously remodels the fibrotic tumor microenvironment and potentiates antitumor immunity. RPm@CC‐LY achieves efficient tumor accumulation and effectively attenuates fibrosis through sustained release of Transforming growth factor‐β (TGF‐β) inhibitor LY2157299 (LY). Concurrently, intracellular acid‐triggered degradation induces Cu^+^‐mediated Fenton‐like reactions and Ca^2+^ overload, leading to oxidative stress, mitochondrial dysfunction, and tumor cell apoptosis. In parallel, RPm@CC‐LY reprograms tumor‐associated macrophages (TAMs) toward an M1 phenotype and enhances dendritic cell maturation, thereby promoting robust CD8^+^ T cell infiltration. Notably, when combined with anti–PD‐1 (αPD1) therapy, RPm@CC‐LY induces a systemic immune response in mice. This work establishes a synergistic nanotherapeutic strategy that integrates fibrosis remodeling with ion interference therapy to overcome the immunotherapeutic barriers posed by fibrosis in PDAC.

## Graphical abstract

A biomimetic nanoplatform (RPm@CC‐LY) remodels fibrotic stroma via sustained TGF‐β inhibition, triggers oxidative stress and apoptosis through Cu^+^‐mediated Fenton chemistry and Ca^2+^ overload, and reprograms macrophages and dendritic cells to boost CD8^+^ T cell infiltration. Combined with anti‐PD‐1, it elicits systemic antitumor immunity, offering a synergistic strategy to overcome immunotherapy resistance in pancreatic cancer.

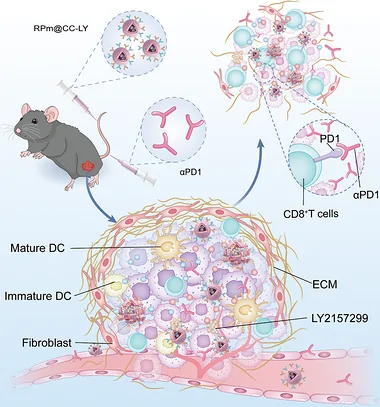

## 1 Introduction

PDAC is characterized by an extremely poor prognosis attributed to its highly desmoplastic and immunosuppressive tumor microenvironment (TME) [1, 2]. The abundant extracellular matrix (ECM) and activated cancer‐associated fibroblasts (CAFs) not only facilitate tumor progression but also form a physical and biochemical barrier that severely limits immune cell infiltration and drug delivery [3, 4, 5]. Consequently, this barrier severely impedes the infiltration of cytotoxic CD8^+^ T cells and the penetration of therapeutic agents. Although immune checkpoint blockade (ICB) therapies targeting PD‐1/PD‐L1 have revolutionized the treatment of multiple cancers [6, 7, 8, 9, 10], their efficacy in PDAC remains minimal [11]. This highlights the critical need to modulate the fibrotic TME to enhance immune cell infiltration and drug delivery [12, 13, 14, 15]. TGF‐β, as a master regulator of fibrotic matrix deposition and tumor immune evasion, has emerged as a critical therapeutic target, directly compromising antitumor immunity by restricting T‐cell infiltration [16, 17, 18, 19, 20, 21]. Preclinical studies demonstrate that co‐inhibition of TGF‐β signaling (e.g., using the small‐molecule inhibitor LY) [22, 23] and PD‐L1 can remodel the TME, reduce ECM deposition, promote CD8^+^ T cell infiltration, and re‐educate macrophages toward an M1 phenotype, leading to synergistic antitumor effects [24, 25, 26, 27]. To effectively deliver such combination therapies to the tumor core, advanced drug delivery systems are essential. Cell membrane coating technology represents a promising biomimetic strategy [28]. By cloaking nanoparticles with natural cell membranes, such as macrophage membranes for immune evasion and prolonged circulation, or tumor cell membranes for homologous targeting, this approach significantly enhances biocompatibility and tumor‐specific accumulation [29, 30, 31, 32, 33, 34, 35].

Ion interference therapy has emerged as an advanced tumor treatment strategy that disrupts cellular homeostasis by modulating metal ion activity. Its primary mechanisms include the induction of oxidative stress, mitochondrial dysfunction, and metabolic interference [36]. Among the various metal ions with therapeutic potential, copper and calcium are particularly notable due to their unique biological roles [37]. Copper ions, leveraging dynamic Cu^+^/Cu^2+^ cycling, catalyze excess hydrogen peroxide in situ within the tumor microenvironment to generate highly toxic hydroxyl radicals, thereby facilitating chemodynamic therapy (CDT) [38, 39, 40]. Concurrently, cell death mediated by copper‐based nanocarriers (cuproptosis) offers novel avenues for overcoming traditional drug resistance [40, 41, 42, 43]. Calcium ions, functioning as crucial intracellular second messengers, are tightly regulated by the endoplasmic reticulum (ER) and mitochondria. As the ER serves as the primary Ca^2+^ reservoir, oxidative stress or the delivery of exogenous Ca^2+^can compromise the ER membrane, triggering the release of stored calcium and leading to cytoplasmic Ca^2+^ overload [44, 45]. Consequently, a synergistic Cu^+^/Ca^2+^ dual overload strategy demonstrates significant antitumor potential by collapsing mitochondrial homeostasis [46].

This study designed a multifunctional biomimetic nanoplatform that primes the tumor microenvironment by disrupting fibrotic barriers, which paves the way for and synergizes with subsequent αPD1 therapy to amplify a potent systemic antitumor immune response. The system consists of hollow mesoporous calcium‐doped copper sulfide, which serves as a carrier, loaded with a TGF‐β inhibitor (LY2157299) and coated with hybrid cell membranes derived from RAW264.7 and Panc02 cells (RPm), collectively termed RPm@CC‐LY. As depicted in Scheme 1, the RPm@CC‐LY nanoplatform employed in this synergistic treatment strategy offers the following specific advantages: (1) Based on the homophilic binding capacity of the hybridized cell membranes, RPm@CC‐LY demonstrates selective tumor accumulation, enabling efficient delivery of LY to tumor sites and enhancing the specificity of immune‐modulating, tumor‐catalyzed therapy. (2) Hollow mesoporous calcium‐doped copper sulfide enhances the LY loading efficiency. The released LY inhibits tumor fibrosis, thereby paving the way for promoting T‐lymphoma cell infiltration. (3) Under tumor acidic conditions, released Cu^2+^ is reduced to Cu^+^, triggering a Fenton‐like reaction that converts H_2_O_2_ into tumor‐toxic •OH. Simultaneously, excess Cu^+^ provokes proteotoxic stress and copper‐mediated cell death. Additionally, released Ca^2+^ disturbs intracellular homeostasis, causing calcium overload and mitochondrial dysfunction that culminates in apoptosis. (4) Induce M2‐to‐M1 polarization of macrophages, promote dendritic cell maturation and antigen presentation, activate systemic immune responses, and effectively enhance CD8^+^ T cell infiltration into tumors. When combined with αPD1 therapy, this synergistic approach generates potent systemic antitumor responses [47, 48, 49, 50]. This effectively prevents tumor recurrence, significantly improving treatment efficacy and long‐term prognosis. In summary, our designed nanotherapeutic strategy, integrating ion interference therapy with immune microenvironment modulation, offers a highly promising approach to overcoming fibrosis‐driven immunotherapy in pancreatic ductal adenocarcinoma.

**SCHEME 1:**
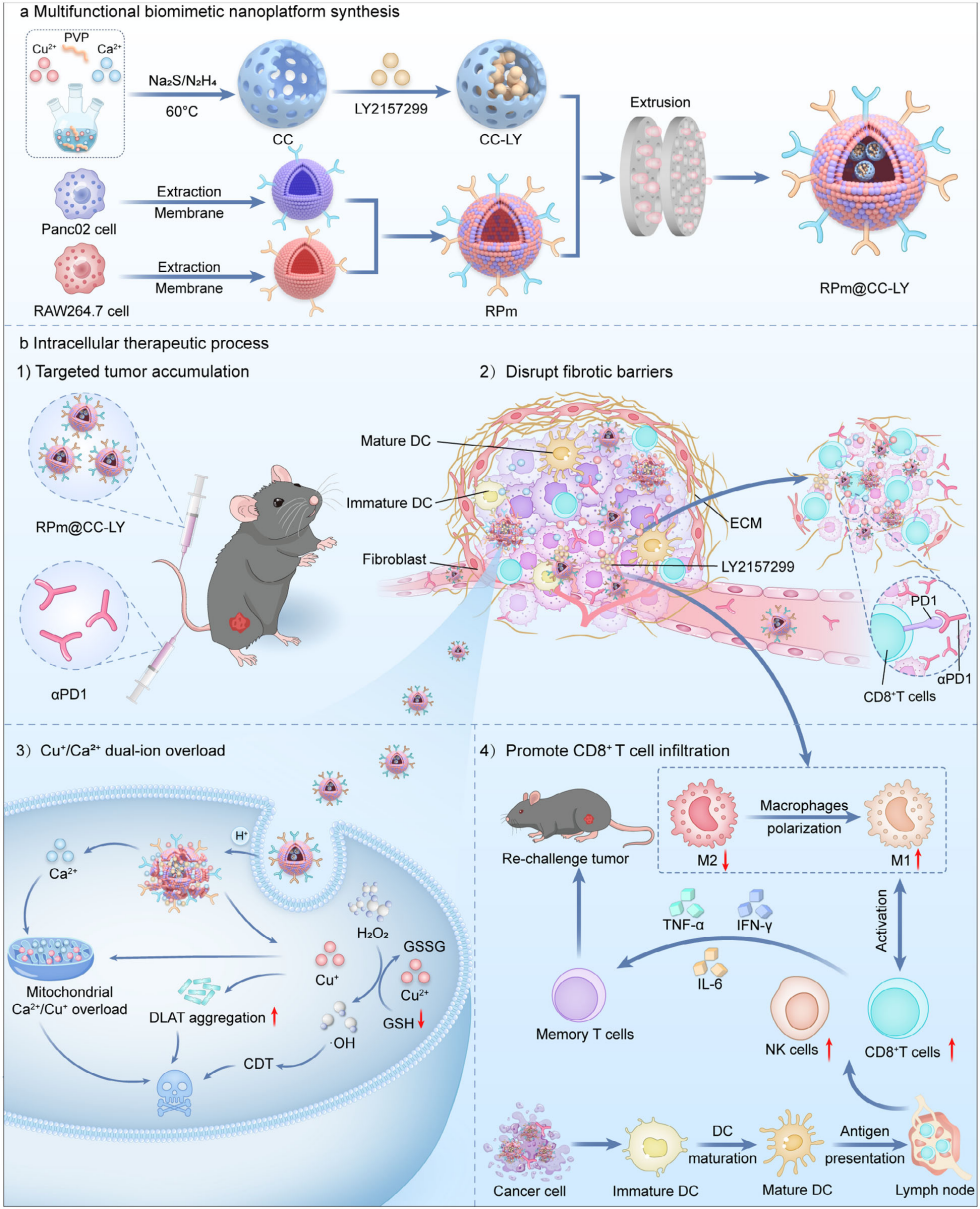
Schematic illustration showing the fabrication process and antitumor mechanism of the multifunctional biomimetic nanoplatform RPm@CC‐LY.

## 2 Results and Discussion

CC nanoparticles were synthesized via a one‐pot method based on Kirkendall diffusion [51, 52]. Transmission electron microscopy (TEM) images illustrate that CC showcases well‐preserved spherical structures with an average diameter of around 109.5 ± 1.22 nm (Figure 1a and Figure S1). High‐resolution TEM images displayed well‐defined crystal planes of CuS clusters within the CC nanoparticles, with clear views along the (101) and (102) planes that precisely correspond to the expected interplanar distances (Figure 1b). Following drug loading, the average hydrodynamic diameter of CC‐LY increased to 147.8 ± 6.35 nm (Figure S2). The X‐ray diffraction (XRD) spectrum of the synthesized CC matched the characteristic peaks of standard CuS, confirming successful synthesis (Figure 1c). Energy‐dispersive spectroscopy (EDS) mappings and spectra demonstrate the existence of Cu, S, and Ca elements (Figure 1d). Additionally, X‐ray photoelectron spectroscopy (XPS) was utilized to further analyze the elemental composition of CC and determine the valence state of copper atoms. The analysis of the full spectrum (Figure S3) revealed the presence of Cu, S, and Ca in CC, consistent with the EDS and mapping results. In the high‐resolution Cu 2*p* spectrum, peaks at 931.81 and 951.60 eV corresponded to Cu^+^, while peaks at 933.89 and 953.47 eV belonged to Cu^2+^ [53], indicating the presence of copper ions in the divalent state within CC. In the S 2*p* spectrum, CuS exhibits characteristic peaks at the S 2*p*3/2 (161.9 eV) and S 2*p*1/2 (169.8 eV) positions corresponding to S^2−^ [54]. The characteristic peaks at 347.7 and 351.2 eV are related to the 2*p*3/2 and 2*p*1/2 orbitals of Ca, respectively (Figure 1e–g).

**FIGURE 1:**
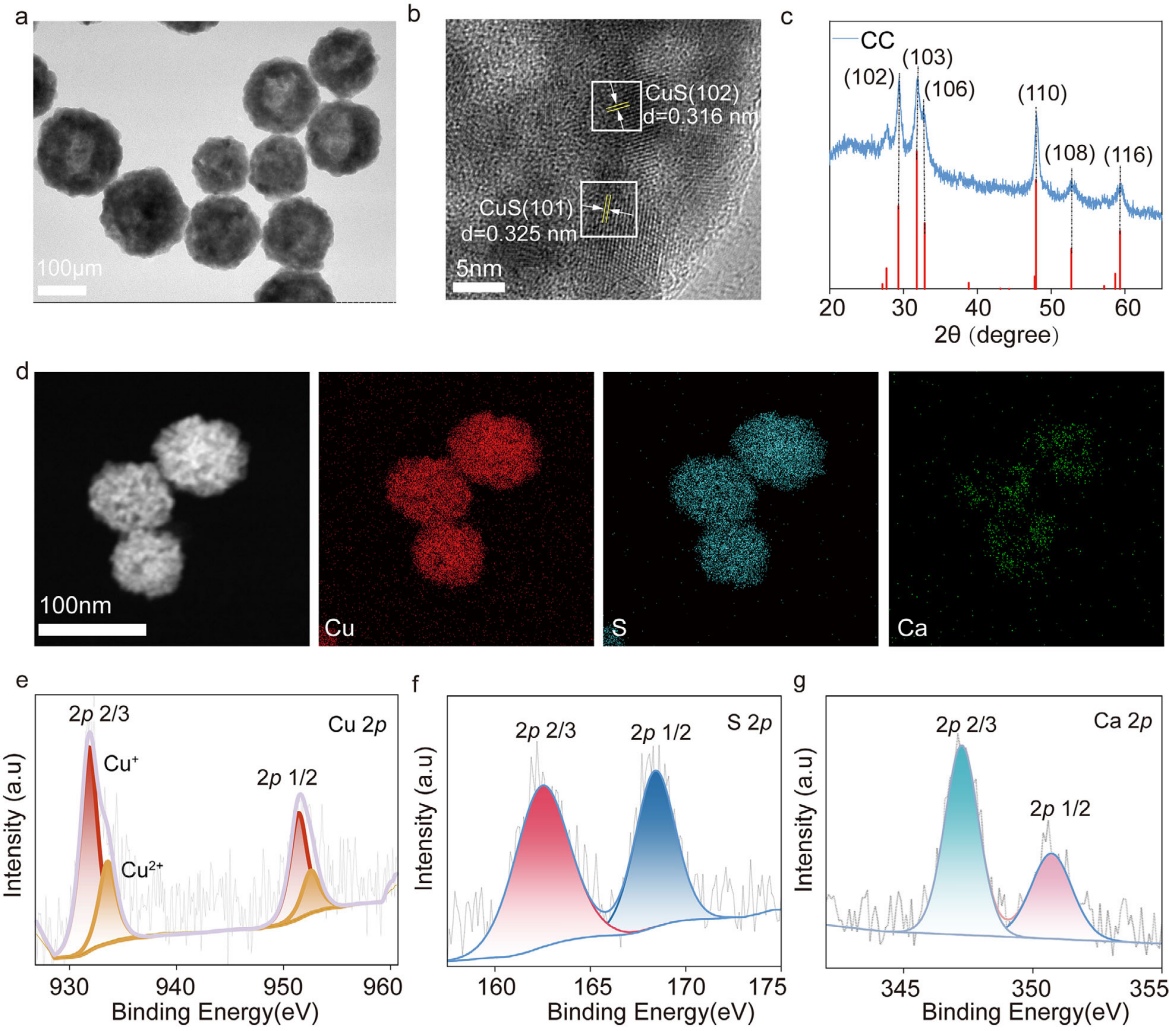
Characterization of CC. (a) TEM image of CC nanoparticles. (b) HR‐TEM image of CC. Scale bar: 5 nm. (c) XRD patterns of CC nanoparticles. (d) EDS mapping of CC nanoparticles. (e) XPS spectrum of Cu 2p in CC. (f) XPS spectrum of S 2p in CC. (g) XPS spectrum of Ca 2p in CC.

This multifunctional biomimetic nanoplatform consists of an LY‐loaded hollow mesoporous calcium‐doped copper sulfide core coated with a hybrid RAW264.7/Panc02 cell membrane. TEM images of RPm@CC‐LY reveal a uniform hybrid film coating the spherical CC‐LY surface (Figure 2a). Furthermore, sodium dodecyl sulfate‐polyacrylamide gel electrophoresis (SDS‐PAGE) was used to examine the membrane protein. The SDS‐PAGE assay showed that the protein composition of RPm@CC‐LY resembled that of RAW264.7, Panc02, and Panc01 cells (Figure 2b and Figure S4). In the dense fibrotic stroma of PDAC, the EPR effect is often counteracted by high interstitial pressure [25], while short peptides are rapidly cleared from the body and carry a risk of off‐target effects [37]. The hybrid cell membrane‐mimicking coating (RPm) endows nanoparticles with dual biointerface properties: “immune evasion” and “homing”. Dynamic light scattering (DLS) measurements show that RPm@CC‐LY has an average hydrodynamic diameter of 186.6 ± 8.66 nm (Figure S5). Furthermore, the surface zeta potential decreased from −8.2 to −22.9 mV after coating with the hybrid film (Figure S6), confirming the presence of a negatively charged outer layer. To further demonstrate successful LY loading, UV–vis absorption spectroscopy was conducted. As shown in Figure 2c, RPm@CC‐LY exhibits a distinct absorption peak at approximately 239 nm, attributed to the characteristic peak of LY, confirming the presence of LY within the RPm@CC‐LY multifunctional biomimetic nanoplatform. Based on the LY calibration curve (Figure S7), the drug loading efficiency of RPm@CC‐LY was calculated to be 19.3%. Concurrently, the N_2_ adsorption–desorption isotherm curves (Figure 2d) and pore size distribution plots (Figures S8 and S9) indicate that the specific surface area of CC decreased from 2.7436 to 2.2453 m^2^g^−1^, and the average pore size decreased from 7.2982 to 6.4499 nm after hybrid membrane coating. Moreover, the RPm@CC‐LY multifunctional biomimetic nanoplatform maintained good stability in different media, with no significant changes in particle size over 3 days (Figure 2e). Notably, at pH 6.5, the structure of RPm@CC‐LY rapidly disintegrated within 24 h (Figure 2f,g), confirming its sensitive acid‐responsive drug release properties within the TME. Figure 2h and Figure S10 demonstrate that over 96 h, free drug LY and CC‐LY released 79.3 ± 0.15% and 73.8 ± 0.45% of LY, respectively, whereas only 38.37 ± 0.45% of the drug was released from RPm@CC‐LY. Furthermore, at the acidic pH values of 5.0 and 6.5, loaded LY released 51.93 ± 0.55% and 42.67 ± 0.28% from RPm@CC‐LY, respectively, indicating that the hybrid membrane coating effectively prevents off‐target and premature LY release by enabling selective disintegration within the acidic TME [55]. Next, we tested its enzymatic activity. Hydroxyl radical (•OH) generation was detected using 3,3ʹ,5,5ʹ‐tetramethylbenzidine (TMB) as a probe. TMB undergoes oxidation by highly reactive •OH to form ox‐TMB, which exhibits characteristic absorption peaks at 370 and 652 nm (Figure 2i). Furthermore, we validated the superior hydroxyl radical generation capability of RPm@CC‐LY via the MB assay. As shown in Figure S11, both CC and RPm@CC‐LY significantly reduced the absorption of methylene blue (MB) at 665 nm, further corroborating •OH generation. Glutathione (GSH), highly expressed in tumor cells, acts as an endogenous antioxidant to counter oxidative stress and maintain redox homeostasis [56]. The potential of RPm@CC‐LY as a GSH scavenger was analyzed using 5,5'‐dithiobis (2‐nitrobenzoic acid) (DTNB) as a probe. As depicted in Figure 2j, RPm@CC‐LY rapidly depletes protective GSH. All these results convincingly demonstrate that the multifunctional biomimetic nanoplatform RPm@CC‐LY possesses the ability to generate hydroxyl radicals and consume GSH, thereby laying the foundation for disrupting intracellular redox homeostasis and subsequent tumor treatment.

**FIGURE 2:**
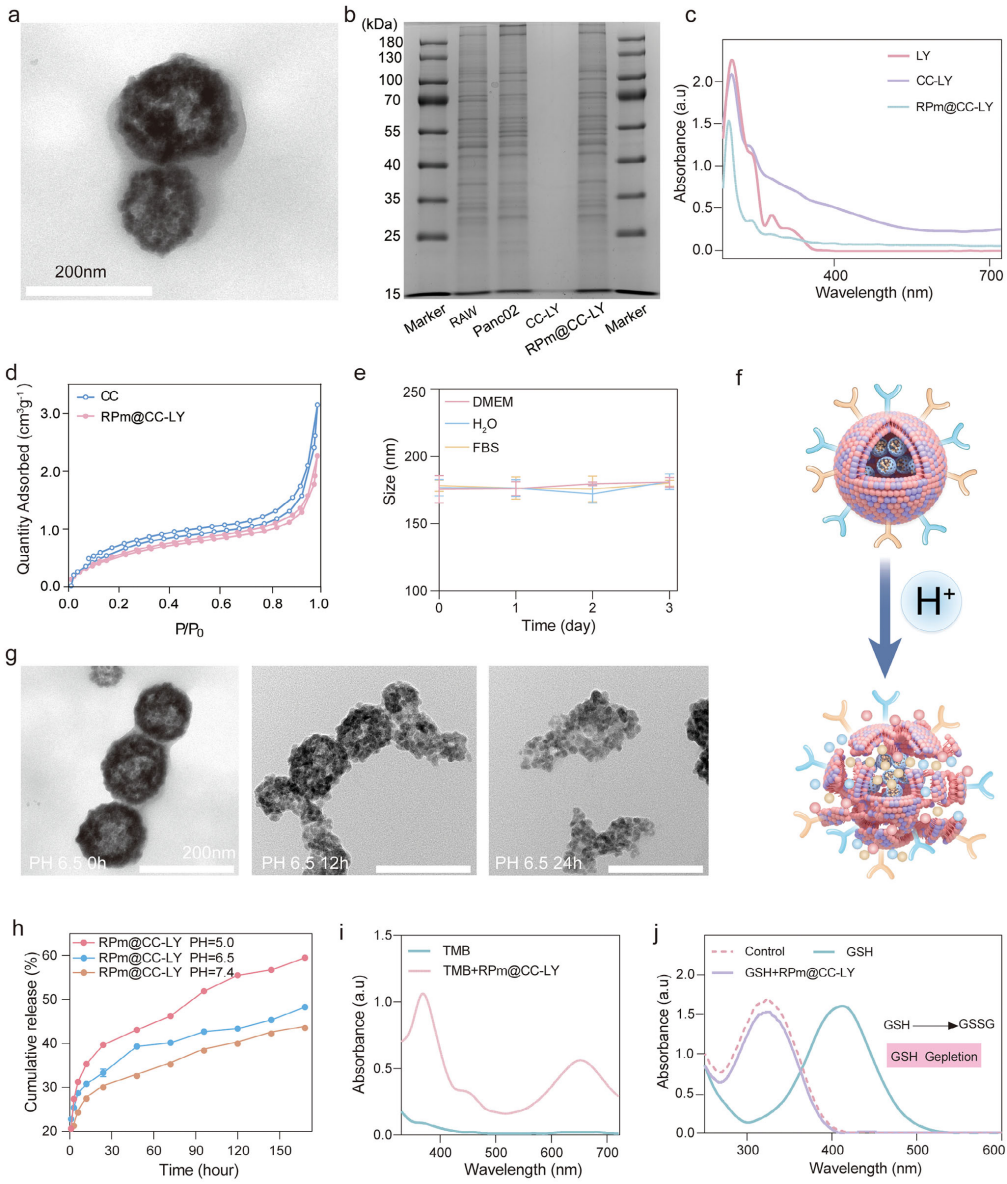
Characterization of the RPm@CC‐LY multifunctional biomimetic nanoplatform. (a) TEM images of the RPm@CC‐LY. (b) SDS‐PAGE protein analysis of RAW264.7, Panc02, CC‐LY, and RPm@CC‐LY, visualized by Coomassie blue staining. (c) UV–vis absorption spectra of free LY, CC‐LY, and RPm@CC‐LY solutions. (d) Nitrogen (N_2_) adsorption–desorption isotherms for CC and RPm@CC‐LY. (e) Particle size of RPm@CC‐LY in DMEM, H2O, and fetal bovine serum (FBS) at 4°C. (f) Schematic diagram illustrating disintegration of the RPm@CC‐LY Multifunctional biomimetic nanoplatform. (g) TEM images of RPm@CC‐LY incubated in PBS solution (pH 6.5) for 0, 12, and 24 h. (h) In vitro release profiles of RPm@CC‐LY in PBS at pH 5.0, 6.5, and 7.4 (*n* = 3). (i) Generation of •OH. (j) Depletion of GSH.

The RPm@CC‐LY multifunctional biomimetic nanoplatform effectively generates •OH radicals and releases Cu^2+^ and Ca^2+^ ions. Given these properties, we investigated the biological efficacy and mechanisms of RPm@CC‐LY against tumor cells (Figure 3a). First, we evaluated the biocompatibility and cytotoxic efficacy of RPm@CC‐LY against Panc01 and Panc02 cells using the 3‐(4,5‐dimethylthiazol‐2‐yl)‐2,5‐diphenyl‐4‐bromotiazol‐9‐yl‐tetrazolium chloride (MTT) assay. To assess the biocompatibility of our multifunctional biomimetic nanoplatform, we first measured the viability of normal liver cells (AML12) after incubation with CC, CC‐LY, and RPm@CC‐LY. Both CC and RPm@CC‐LY exhibited minimal effects on cell proliferation compared to LY (Figures S12 and S13). Similarly, apoptosis rates followed the same trend, supporting the non‐cytotoxic nature of CC and confirming its safety as a drug carrier (Figures S14 and S15). Furthermore, CC‐LY and RPm@CC‐LY displayed greater cytotoxicity toward Panc01 and Panc02 cells than LY alone (Figures S16–S19). As shown in Figure 3b,c, the apoptosis rates of Panc02 cells treated with CC‐LY (45.84%) or RPm@CC‐LY (47.21%) were significantly higher than those in the control group (5.93%, *p* < 0.0001) and the free LY group (22.79%, *p* < 0.0001). A similar pattern was observed in Panc01 cells (Figures S20 and S21). This may be due to the hybrid membrane‐coated nanoscale system exhibiting superior internalization and sustained release behavior. Live/dead cell staining showed no detectable cell death (red fluorescence) in the control group, whereas CC‐LY and RPm@CC‐LY treatments led to significantly greater cell death compared to free LY and CC (Figure 3d and Figure S22). Scratch assay results indicated that all nanodrugs significantly inhibited migration in both Panc01 and Panc02 cells compared to the control (Figures S23–S26). In addition, RPm@CC‐LY/NR exhibited higher cellular uptake efficiency than free NR in both cell lines (Figure 3e,f and Figures S27 and S28). The 2',7'‐dichlorofluorescein diacetate (DCFH‐DA) probe was used to detect intracellular •OH radicals. Fluorescence intensity in Panc01 and Panc02 cells treated with CC, CC‐LY, and RPm@CC‐LY was markedly higher than in the controls, suggesting that Cu^+^ derived from CuS decomposition facilitated a Fenton‐like reaction with H_2_O_2_, generating substantial •OH radicals and causing a surge in exogenous reactive oxygen species (ROS) (Figure 3g and Figure S29). We also assessed mitochondrial membrane potential (MMP) using the JC‐1 probe. On intact mitochondrial membranes, JC‐1 aggregates emit red fluorescence, while depolarization leads to green fluorescence emission (Figure 3h and Figure S30). Notably, RPm@CC‐LY treatment resulted in strong green fluorescence signals in tumor cells, indicating a significant loss of MMP, potentially due to copper‐calcium ion dual overload. Correspondingly, RPm@CC‐LY induced higher levels of DNA damage compared to the control group (Figure 3i and Figure S31). Generally, ROS can alter protein conformation and desensitize calcium channels and pumps, leading to uncontrolled intracellular Ca^2+^ accumulation [37]. Increased Ca^2+^ ion levels within tumor cells were confirmed by Ca^2+^ fluorescence imaging and alizarin red staining (Figure 3j,k and Figures S32 and S33). This may lead to calcium overload and mitochondrial dysfunction, ultimately triggering apoptosis. Following treatment with RPm@CC‐LY, the expression of FDX1 and LIAS proteins in tumor cells was significantly downregulated, while a substantial accumulation of high‐molecular‐weight DLAT oligomers was observed. These results demonstrate that the nanoparticles can induce specific copper‐mediated cell death(Figure S34). Compared with photodynamic therapy (PDT) [25], ion‐based therapeutic strategies demonstrate unique advantages in this system; the hypoxic microenvironment deep within pancreatic tumors severely quenches the singlet oxygen generated by PDT, thereby greatly limiting its efficacy, whereas ion therapy is not constrained by these oxygenation levels. Furthermore, we evaluated the effects of RPm@CC‐LY on mouse bone marrow‐derived dendritic cells (BMDCs). As shown in Figure 3l–n, RPm@CC‐LY demonstrated a strong ability to promote dendritic cell maturation. The sustained production of ROS effectively promoted the maturation of dendritic cells and antigen cross‐presentation, activated antitumor immunity, and significantly enhanced antitumor efficacy [57].

**FIGURE 3:**
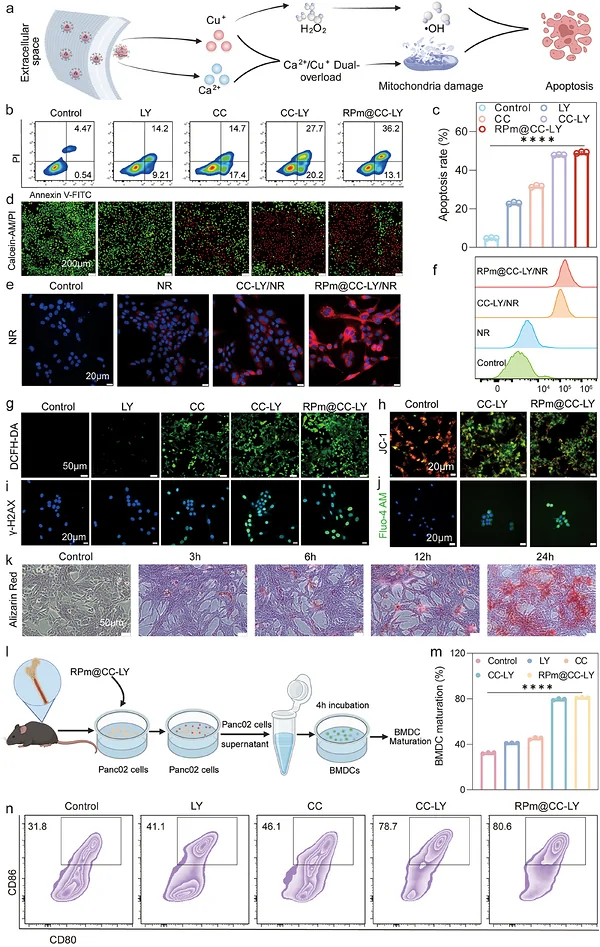
In vitro therapeutic efficacy of synergistic therapy. (a) Mechanism of RPm@CC‐LY‐induced cell apoptosis. (b) Flow cytometric analysis of the Annexin V‐FITC/PI‐stained Panc02 cells in different groups; (c) Apoptosis rate in different groups. (d) Fluorescence images of the live and dead Panc02 cells in different groups. (e) Fluorescence images of different drugs taken up by Panc02 cells. (f) Flow cytometry analysis of different drug uptake by Panc02 cells. (g) Fluorescence images of DCFH‐DA‐stained Panc02 cells after treatment with various drugs for 24 h. (h) Fluorescence images of the activity in Panc02 cells of mitochondria under various treatments. Scale bar = 20 µm. (i) Fluorescence images of the expression levels of γ‐H2AX in Panc02 cells in different groups. (j) Intracellular Ca^2+^ ions’ accumulation in Panc02 cells. (k) Panc02 cells were incubated with RPm@CC‐LY for varying periods and then stained with alizarin red to identify in vitro cellular products that exhibited red areas of calcification. (l) Flowchart of experiments related to RPm@CC‐LY action on murine bone marrow‐derived dendritic cells. (m) BMDCs maturation in each group. (n) Flow cytometry analysis of CD80^+^ and CD86^+^ expression by BMDCs after incubation with LY, CC, CC‐LY and RPm@CC‐LY. Results are presented as means ± SD. Statistical significance was determined using one‐way ANOVA for multiple group comparisons, followed by Tukey's two‐tailed post‐hoc test for pairwise analysis; all tests were two‐sided. Significance thresholds: ^*^ *p* < 0.05, ^**^ *p* < 0.01, ^***^ *p* < 0.001, ^****^ *p* < 0.0001.

Intravenous injection of indocyanine green (ICG)‐labeled RPm@CC‐LY was performed to investigate its in vivo biodistribution. As shown in Figure 4a, free ICG was gradually cleared over time, whereas RPm@CC‐LY exhibited persistent accumulation in tumor tissue up to 48 h post‐injection, confirming its superior active targeting capability. Ex vivo imaging of tumor tissue at 48 h (Figure 4b) also revealed intense fluorescence signals. Additionally, transcriptomic analysis was conducted to comprehensively evaluate the effects of RPm@CC‐LY combined with αPD1 treatment on gene expression in Panc02 cells. Principal component analysis (PCA) revealed significant differences in gene expression profiles between the RPm@CC‐LY+αPD1 group and the control group (Figure 4c). A volcano plot illustrated the overall distribution of differentially expressed genes (DEGs) between these groups (Figure 4d). In total, we identified 1,043 DEGs, comprising 505 downregulated and 538 upregulated genes. These substantial gene expression changes suggest that RPm@CC‐LY+αPD1 exerts distinct molecular effects on cancer progression. Notably, Gene Ontology (GO) bubble plot analysis revealed that the genes regulated by RPm@CC‐LY+αPD1 were predominantly associated with genomic regions encoding dense ECM components (Figure 4e). Furthermore, KEGG pathway enrichment analysis indicated that RPm@CC‐LY+αPD1 significantly influences key signaling pathways, such as cytokine‐cytokine receptor interactions and transcriptional dysregulation in cancer (Figure 4f). Gene set enrichment analysis (GSEA) further demonstrated activation of pathways related to immune responses, T cell receptor signaling, and cytokine‐cytokine receptor interactions. Collectively, these findings highlight that RPm@CC‐LY+αPD1 induces potent immunomodulatory effects through multiple pathways, effectively promoting apoptosis and remodeling the tumor microenvironment to enhance the efficacy of cancer immunotherapy (Figure 4g–i).

**FIGURE 4:**
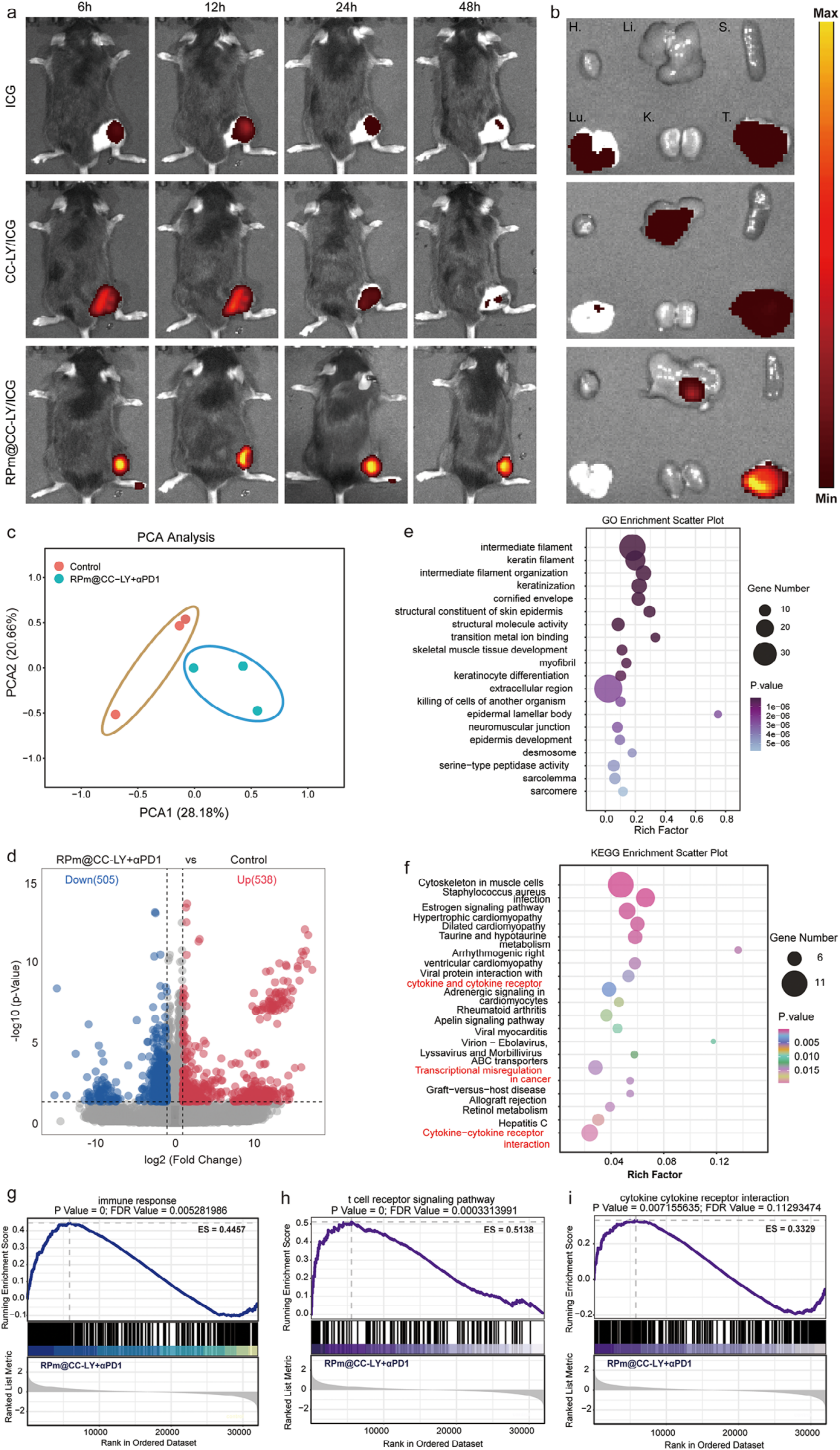
RPm@CC‐LY multifunctional biomimetic nanoplatform targeting tumor tissue and mechanism exploration. (a) Fluorescence images of Panc02 tumor‐bearing mice following intravenous injection of free ICG, CC‐LY/ICG, and RPm@CC‐LY/ICG. (b) Ex vivo fluorescence images of isolated organs and tumors at 48 h post‐injection (H: heart; Li: liver; S: spleen; Lu: lung; K: kidney; T: tumor). (c) PCA plot showing differences in gene expression between the control group and the RPm@CC‐LY+αPD1 treatment group (*n* = 3). (d) Volcano plot of DEGs between the RPm@CC‐LY+αPD1 and control cohorts. Significant DEGs are indicated by red and blue dots. (e) GO enrichment analysis of DEGs (*n* = 3). (f) KEGG pathway enrichment analysis of signaling pathways altered by RPm@CC‐LY+αPD1 treatment. (g–i) GSEA showing significantly enriched pathways related to the immune response (g), T cell receptor signaling (h), and cytokine–cytokine receptor interactions (i) in the RPm@CC‐LY+αPD1 group compared to the control.

To evaluate the antitumor efficacy of RPm@CC‐LY+αPD1 in vivo, the biomimetic nanomedicine was administered intravenously to Panc02 tumor‐bearing mice (Figure 5a). Compared with other groups, the RPm@CC‐LY+αPD1 group exhibits the most significant inhibitory effect on tumor growth (Figure 5b–d and Figures S35–S42). No significant changes in body weight were observed across all groups during the treatment period (Figure 5e and Figure S43). Furthermore, the RPm@CC‐LY+αPD1 group demonstrated a significantly prolonged survival time in the tumor‐bearing mice (Figure 5f and Figure S44). Notably, hematoxylin and eosin (HE) staining of tumors from the RPm@CC‐LY+αPD1 group showed the most severe cellular damage, characterized by large cavities and extensive cytoplasmic fragmentation within the tumor tissue. Studies indicate that TGF‐β1‐induced activation of CAFs and tumor fibrosis confine T cells within the stroma, preventing them from aggregating around cancer cells due to the dense ECM and thereby inducing intrinsic immune resistance. However, reversing fibroblast activation using the TGFR1 inhibitor LY alleviates the intrinsic immune resistance caused by activated TGF‐β signaling pathways [58]. Immunohistochemical analysis revealed a significant reduction in pSmad3 levels in the RPm@CC LY+αPD1 group, suggesting that RPm@CC LY effectively blocked the TGF‐β/Smad signaling pathway within the tumor microenvironment [25]. Furthermore, the combination treatment significantly downregulated the expression of α‐SMA, FAP, and fibronectin, and reduced collagen deposition. These findings indicate that RPm@CC LY+αPD1 reversed CAF activation, disrupted the ECM barrier, and facilitated T‐cell migration in vivo (Figures S45–S49). Collagen I/CD3 co‐staining further showed a decrease in collagen I levels accompanied by increased CD3^+^ T‐cell infiltration (Figure S50), supporting an association between stromal remodeling and enhanced T‐cell infiltration in vivo. Additionally, fluorescence images from terminal deoxynucleotidyl transferase dUTP nick‐end labeling (TUNEL) assays revealed a stronger green fluorescence in the RPm@CC‐LY+αPD1 group, indicating enhanced tumor cell apoptosis. To assess the in vivo induction of copper‐mediated cell death, DLAT expression in mouse tumor tissues was detected via immunofluorescence staining, showing the strongest red fluorescence in the RPm@CC‐LY+αPD1 group, indicating that Cu^2+^ overload induced DLAT aggregation, thereby triggering copper‐mediated tumor cell death (Figure 5g). Concurrently, Ki‐67 analysis demonstrated that RPm@CC‐LY+αPD1 treatment significantly inhibited tumor cell proliferation (Figure S51). The combination therapy of RPm@CC‐LY and αPD1 achieves maximal tumor suppression by reversing tumor fibrosis via TGF‐β signaling pathway inhibition and synergistically inducing cell death through ion interference. The biosafety of RPm@CC‐LY+αPD1 was further evaluated through in vitro hemolysis assays, H&E staining of major organs, and hematological analysis. No significant hemolysis was observed with any of the LY, CC, CC‐LY, or RPm@CC‐LY formulations (Figure S52). Images of red blood cells (RBCs) (Figure S52a), hemolysis (Figure S52b), UV–vis absorption spectra (Figure S52c), and hemoglobin concentration (Figure S52d) further demonstrated the excellent hematological compatibility of RPm@CC‐LY+αPD1. HE staining of major organs (heart, liver, spleen, lung, and kidney) revealed no detectable toxicity following various treatments (Figure 5h and Figure S53). Furthermore, no significant differences in hematological parameters were observed in the RPm@CC‐LY+αPD1 group after multiple treatments compared to the control group (Figure 5i and Figure S54). These results further demonstrate the satisfactory biocompatibility of the multifunctional biomimetic nanoplatform.

**FIGURE 5:**
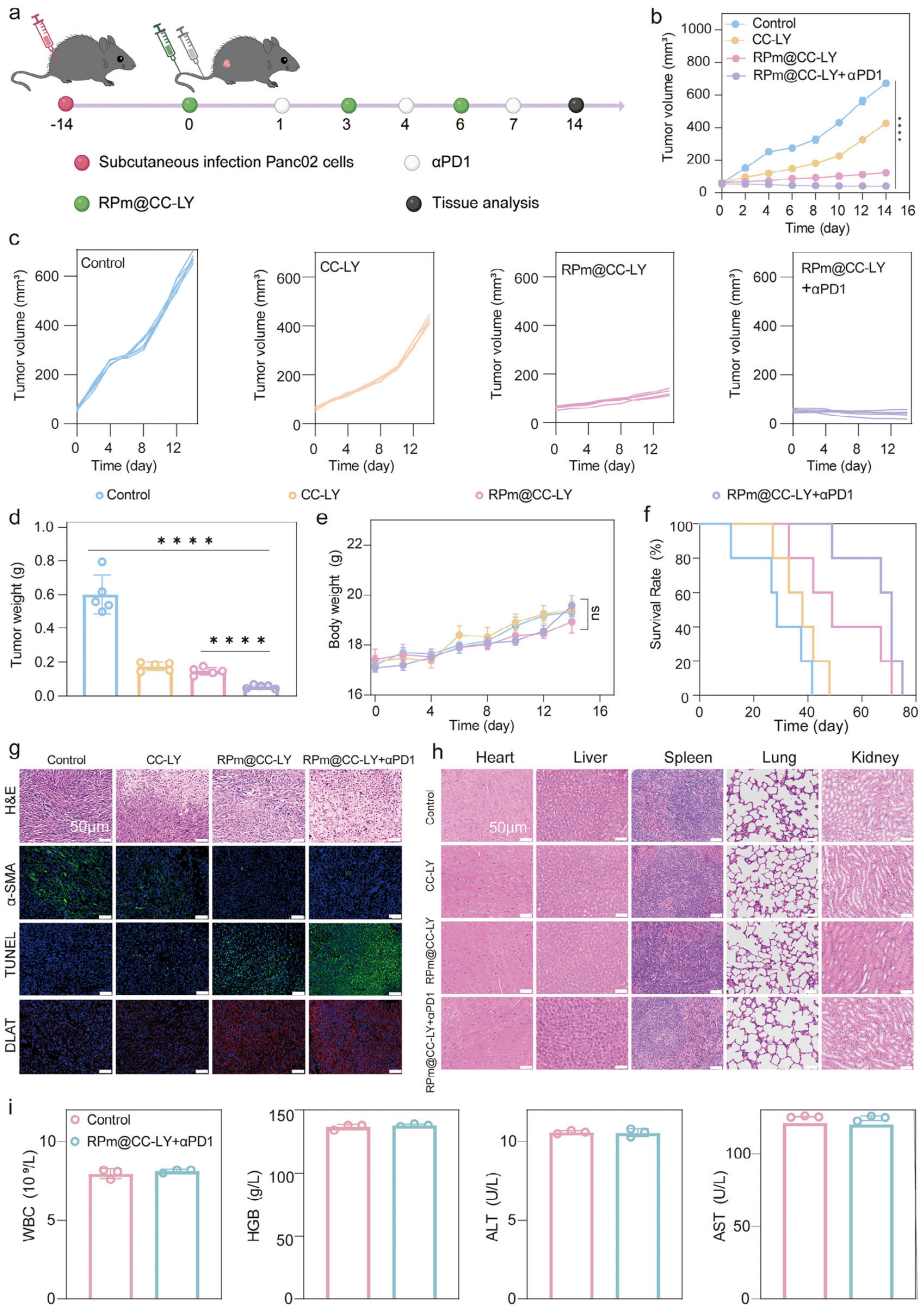
RPm@CC‐LY multifunctional biomimetic nanoplatform exerts in vivo antitumor effects by alleviating tumor fibrosis and promoting CD8^+^ T cell infiltration into tumors. (a) Schematic diagram of the treatment procedure. (b) Tumor growth curves of Panc02 tumor‐bearing mice across different groups (*n* = 6). (c) Individual tumor growth curves for each Panc02 tumor‐bearing mouse in each group (*n* = 6). (d) Tumor weights. (e) Body weight changes during treatment (*n* = 6). (f) Survival curves of each group under different treatments (*n* = 6). (g) H&E staining of tumors following various treatments, as well as immunofluorescence images of α‐SMA, TUNEL, and DLAT in tumor sections (*n* = 3). Scale bar: 50 µm. (h) Representative H&E‐stained images of major organ tissue sections (scale bar: 50 µm). (i) In vivo analysis of systemic toxicity for various treatments, assessed by biochemical markers and routine blood parameters. Results are presented as means ± SD. Statistical significance was determined using the Student's t‐test for two‐group comparisons and one‐way ANOVA for multiple‐group comparisons, followed by Tukey's two‐tailed post‐hoc test for pairwise analysis; all tests were two‐sided. Significance thresholds: ^*^ *p* < 0.05, ^**^ *p* < 0.01, ^***^ *p* < 0.001, ^****^ *p* < 0.0001.

We also confirmed that the enhanced immunomodulatory combination therapy induced notable alterations in the tumor immune microenvironment (Figure 6a). Immunofluorescence staining revealed that CD8^+^ T cells in the RPm@CC‐LY+αPD1 group exhibited the strongest fluorescence signals, confirming that RPm@CC‐LY+αPD1 exerts antitumor effects in vivo by alleviating tumor fibrosis and promoting the infiltration of cytotoxic T lymphocytes (CTL) into tumors (Figure 6b and Figure S55). Dendritic cell maturation plays a crucial role in activating T cells and inducing systemic antitumor immune responses. Notably, the percentage of mature DCs in both tumors, spleen, and lymph nodes increased significantly to 31.2%, 26.1%, and 17.9%, respectively, in the RPm@CC‐LY+αPD1 group, representing approximately 10.87‐fold, 5.09‐fold, and 6.17‐fold increases compared to the PBS group. This demonstrates a robust effect in promoting DC maturation (Figure 6c–e and Figures S56–S61). The quantification of infiltrating CD3^+^CD8^+^ T cells in tumors and spleens also increased markedly after RPm@CC‐LY+αPD1 treatment, reaching 31.7% and 40.6%, respectively, compared to 3.01% and 26.2% in the PBS group (Figure 6f–i and Figures S62–S65), indicating that RPm@CC‐LY+αPD1 has a strong capacity to promote CTL infiltration. Moreover, following RPm@CC‐LY+αPD1 treatment, the proportion of M1‐like macrophages significantly increased in both tumors (Figure 6j–l and Figures S66–S68) and spleens (Figures S69–S71), while M2 macrophage numbers decreased markedly. This suggests that RPm@CC‐LY+αPD1 effectively reprograms TAMs from a pro‐tumorigenic M2 phenotype to an anti‐tumor M1 phenotype. Administration of RPm@CC‐LY+αPD1 also resulted in increased IFN‐γ secretion within tumors, along with significant upregulation of tumor necrosis factor‐α (TNF‐α), providing further evidence of a robust immune response. Concurrently, levels of IL‐6—a key mediator in immune responses—were markedly elevated in the RPm@CC‐LY+αPD1 group (Figure 6m–o and Figures S72–S74). Taken together, these findings demonstrate that RPm@CC‐LY+αPD1 promotes intratumoral infiltration of CD8^+^ T cells by enhancing DC maturation and facilitates the polarization of macrophages from the M2 to the M1 phenotype.

**FIGURE 6:**
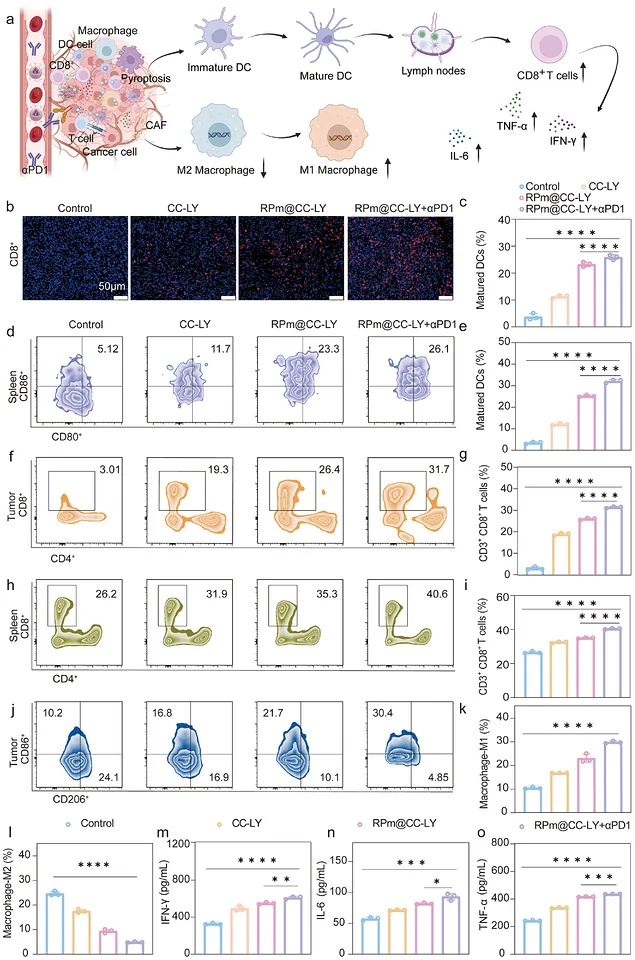
In vivo immune activation of RPm@CC‐LY + αPD1 in Panc02 mouse models. (a) Schematic diagram illustrating the in vivo immune activation effects of RPm@CC‐LY+αPD1. (b) Immunofluorescence images showing CD8^+^ T cells in tumor sections (scale bar = 50 µm). (c, d) Flow cytometric and quantitative analyses of mature dendritic cells (CD80^+^CD86^+^) in the spleen. (e) Quantitative analysis of mature DCs (CD80^+^CD86^+^) in tumor samples from mice. (f, g) Flow cytometric and quantitative analyses of CD8^+^ T cells (CD45^+^CD3^+^CD8^+^) in tumor samples from mice. (h, i) Flow cytometric and quantitative analyses of CD8^+^ T cells in the spleen. (j–l) Flow cytometric and quantitative analyses of CD80^+^CD206^+^ cells in tumor tissues. (m–o) Measurement of IFN‐γ, IL‐6, and TNF‐α levels in serum from each treatment group. Results are presented as means ± SD. Statistical significance was determined using the Student's t‐test for two‐group comparisons and one‐way ANOVA for multiple‐group comparisons, followed by Tukey's two‐tailed post‐hoc test for pairwise analysis; all tests were two‐sided. Significance thresholds: ^*^ *p* < 0.05, ^**^ *p* < 0.01, ^***^ *p* < 0.001, ^****^ *p* < 0.0001.

Our findings indicate that RPm@CC‐LY+αPD1 effectively stimulates CD8^+^ T cells within the TME. Furthermore, RNA sequencing validates the treatment's systemic antitumor activity. Consequently, RPm@CC‐LY+αPD1 holds significant promise as a therapeutic strategy. To investigate whether RPm@CC‐LY+αPD1 can induce a durable systemic immune response, we conducted a tumor challenge assay (Figure 7a). Specifically, we established a Panc02 pancreatic cancer model and divided the animals into six groups. After the end of treatment, Panc02 cells were injected into the opposite thigh of C57BL/6 mice to serve as a re‐challenge tumor. Throughout the experiment, we continuously monitored the volumes of both primary and re‐challenge tumors, as well as mouse body weight (Figure 7b,c and Figure S75). Both primary and re‐challenge tumors in the RPm@CC‐LY+αPD1 treatment group were smaller compared to those in the Control, LY+αPD1, and RPm@CC‐LY groups (Figures S76 and S77). Furthermore, the RPm@CC‐LY+αPD1 group exhibited significantly greater suppression of re‐challenge tumor weight relative to the other groups (Figure 7d,e). To investigate the underlying mechanism of RPm@CC‐LY+αPD1‐induced suppression of re‐challenge tumors, we employed flow cytometry. Compared with the other groups, the RPm@CC‐LY+αPD1 group showed a higher proportion of CD3^+^CD8^+^ T cells in both re‐challenge tumors and the spleen (Figure 7f–i). Additionally, the quantification of effector memory T cells (Tem, CD8^+^CD44^+^CD62L^−^) in re‐challenge tumor tissue and spleen increased significantly to 74.7% and 22.0%, respectively, compared to the control group, which showed much lower proportions at 16.1% and 12.5% (Figure 7j,k, Figures S78 and S79). The RPm@CC‐LY+αPD1 group also exhibited an increased proportion of NK cells in both re‐challenge tumors and spleen (Figures S80–S83). After euthanizing the mice, tumors were collected for histological analysis. H&E staining revealed that, compared to the other groups, the RPm@CC‐LY+αPD1 group induced extensive cellular necrosis and tumor destruction in re‐challenge tumors. Simultaneously, Ki‐67 staining showed that RPm@CC‐LY+αPD1 treatment inhibited tumor‐cell proliferation, while the reduced expression of α‐SMA, FAP, and fibronectin indicated attenuation of the fibrotic stroma. Furthermore, RPm@CC‐LY+αPD1 increased the number of TUNEL‐positive apoptotic cells within tumor tissues, suggesting enhanced antitumor efficacy. The RPm@CC‐LY+αPD1 group exhibited the strongest red fluorescence, indicating that it induced DLAT aggregation and triggered copper‐mediated tumor cell death. Additionally, immunofluorescence staining revealed that the RPm@CC‐LY+αPD1 treatment promoted the infiltration of CTL into the tumor (Figure 7l,m and Figures S84 and S85). Notably, mice treated with RPm@CC‐LY+αPD1 exhibited markedly increased secretion levels of TNF‐α, IL‐6, and IFN‐γ in both bilateral tumors (Figure 7n–p), further confirming that RPm@CC‐LY+αPD1 effectively activates robust immune memory responses to eliminate re‐challenge tumors.

**FIGURE 7:**
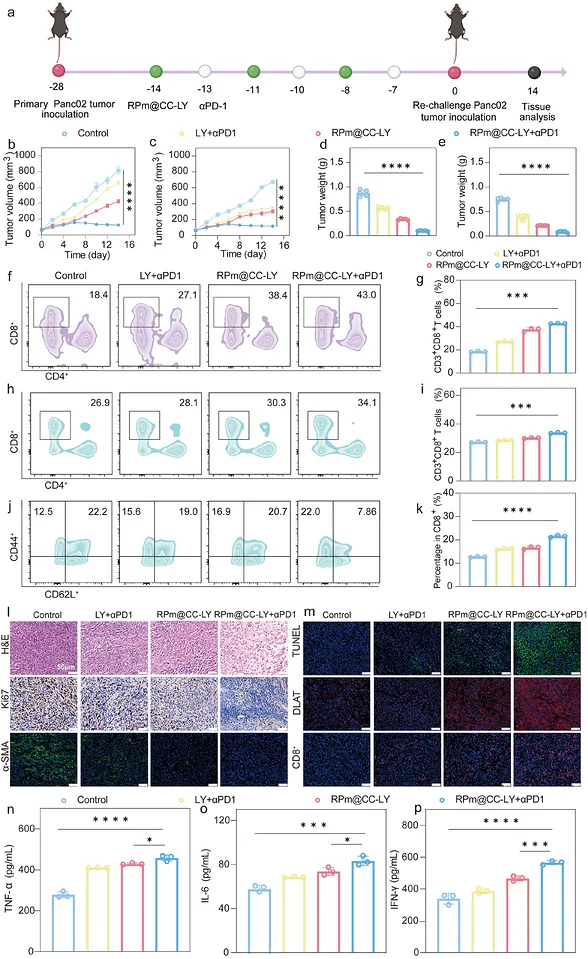
Sustained anti‐tumor immunity in re‐challenge tumor models. (a) Schematic illustration of the experimental timeline for treatment in the subcutaneous Panc02 bilateral tumor model. (b, c) Growth curves of the primary tumor (b) and re‐challenge tumors (c) in mice bearing Panc02 tumors from each treatment group (*n* = 6). (d, e) Average weights of primary tumors (d) and re‐challenge tumors (e) in the different treatment groups after treatment (*n* = 5). (f, g) Representative flow cytometric analysis and quantification of CD8^+^ T cells in re‐challenge tumors. (h, i) Representative flow cytometric analysis and quantification of CD8^+^ T cells in the spleen. (j, k) Representative flow cytometric analysis and quantification of effector memory T cells (Tem, CD44^+^ CD62L^−^) among CD8^+^ T cells in the spleen across different treatment groups. (l) Histological analysis of tumors by H&E, Ki‐67, and α‐SMA staining following treatment (*n* = 3). Scale bar: 50 µm. (m) TUNEL, DLAT, and CD8_+_ T cell immunofluorescence images of tumors after various treatments. (n–p) Serum levels of TNF‐α, IL‐6, and IFN‐γ in each group of mice post‐treatment. Results are presented as means ± SD. Statistical significance was determined using the Student's t‐test for two‐group comparisons and one‐way ANOVA for multiple‐group comparisons, followed by Tukey's two‐tailed post‐hoc test for pairwise analysis; all tests were two‐sided. Significance thresholds: ^*^ *p* < 0.05, ^**^ *p* < 0.01, ^***^ *p* < 0.001, ^****^ *p* < 0.0001.

## 3 Conclusion

In summary, this study reports a multifunctional biomimetic nanoplatform RPm@CC‐LY, designed to address the dual challenges of fibrosis and immunosuppression in PDAC. The platform first achieves homotopic targeting via its hybrid cell membrane coating, facilitating targeted tumor enrichment and potent antitumor activity. The delivered TGF‐β inhibitor, LY, actively remodels the fibrotic matrix to breach critical physical and immunosuppressive barriers. This “pretreatment” of the tumor microenvironment disrupts fibrosis, creating conditions favorable for antitumor immunity. Concurrently, acid‐triggered degradation of the core upon internalization releases Cu^2+^ and Ca^2+^ ions, initiating a cascade of enhanced antitumor effects through a dual‐ion overload mechanism. When combined with αPD1 checkpoint blockade, this sequential strategy synergistically promotes dendritic cell maturation and M1 macrophage polarization, while enhancing intratumoral CD8^+^ T cell infiltration and activation. Consequently, the combination of RPm@CC‐LY and αPD1 not only inhibits primary tumor growth but also elicits systemic antitumor immune responses, effectively blocking tumor recurrence and establishing durable immune memory. Collectively, this study establishes a novel nanotherapeutic paradigm that targets tumors for fibrosis reversal, synergistically inducing ion disruption and immune activation, offering a robust strategy for effective cancer treatment.

## 4 Experimental Section

### 4.1 Materials

CaCl_2_, CuCl_2_, Na_2_S, hydrazine hydrate, PVP‐K30, MB, TMB, LY, and DCFH‐DA were purchased from Shanghai Macklin Biochemical Technology Co. All chemicals were used as received without further purification.

### 4.2 Synthesis of CC

240 mg of PVP‐K30 was dissolved in 25 mL of deionized water. Subsequently, 100 µL of CuCl_2_ (85 mg mL^−1^) and 100 µL of CaCl_2_ (70 mg mL^−1^) were added. The mixture was stirred at 1200 rpm at room temperature for 5 min. Next, 25 mL of NaOH solution (pH 9.0) was added, and after thorough mixing, 8 µL of N_2_H_4_·H_2_O (80%) was added dropwise to the reaction mixture. The solution was magnetically stirred for 5 min, followed by the addition of 200 µL of Na_2_S aqueous solution (320 mg mL^−1^), and the reaction was allowed to proceed at 60°C for 2 h. After completion, the mixture was centrifuged at 13 000 rpm for 10 min. The resulting dark green material was washed three times with deionized water and freeze‐dried for 48 h to obtain CC.

### 4.3 Synthesis of RPm@CC‐LY

Mix Panc01 or Panc02 and RAW 264.7 cell membranes at a 1:1 (w/w) ratio and sonicate for 10 min at 37°C to promote membrane fusion. Slowly add an aqueous solution of CC‐LY (2 mg) to the hybrid membrane solution and mix well. Treat the mixture with a 100 W ultrasonicator for 10 min, then extrude the resulting dispersion repeatedly through polycarbonate membranes with pore sizes of 0.4 and 0.2 µm using a liposome extruder. Purify the final product by centrifuging at 15 000 rpm for 30 min at 4°C, and wash the pellet three times with deionized water. Redisperse the purified biomimetic nanoparticles in deionized water for subsequent experiments.

### 4.4 In Vitro Generation of •OH

The generation of •OH radicals was detected using the TMB assay. Briefly, 1 mL of TMB solution (0.25 mM) was added to 2 mL of CC or RPm@CC‐LY solution (50 µg mL^−1^), followed by stirring for 5 min. The absorbance of TMB was measured at 370 and 652 nm using a UV spectrophotometer. Additionally, an MB solution (1 mL, 20 µM) was utilized as an alternative probe to further validate •OH generation. MB was added to 2 mL of CC or RPm@CC‐LY solution (50 µg mL^−1^) and stirred for 5 min. MB bleaching was assessed by measuring the absorbance at 665 nm via UV spectrophotometry.

### 4.5 In Vitro GSH Depletion

The depletion of GSH by different nanoparticles was detected using a DTNB probe. In brief, 100 µL of CC‐LY or RPm@CC‐LY solution (1 mg mL^−1^) was mixed with 100 µL of GSH (10 mM) in 3.8 mL of distilled water and incubated at 37°C for 30 min. The mixture was then centrifuged at 9000 rpm for 10 min. Subsequently, 600 µL of the supernatant was mixed with 3 mL of DTNB solution (0.02 mg mL^−1^) in Tris‐HCl buffer (pH 6.5), and the absorbance at 412 nm was measured using a UV spectrophotometer.

### 4.6 Intracellular ROS Detection

Panc01 and Panc02 cells were separately incubated with 100 µL of PBS, LY, CC, CC‐LY, or RPm@CC‐LY (equivalent to an LY concentration of 10 µg mL^−1^) for 12 h. After washing three times with PBS, the cells were incubated with DCFH‐DA (10 µM) for 30 min. Fluorescence images were then captured using an Olympus IX83 Live Microscope (Olympus Life Science, Japan).

### 4.7 Measurement of Mitochondrial Membrane Potential

To assess MMP, tumor cells were seeded into 6‐well plates and cultured overnight. The cells were then treated with PBS, LY, CC, CC‐LY, or RPm@CC‐LY for 24 h. Subsequently, JC‐1 dye was added, and the cells were incubated for an additional 30 min. After incubation, the cells were washed three times with saline, and images were acquired using an Olympus IX83 Live Microscope (Olympus Life Science, Japan).

### 4.8 Measurement of Intracellular Ca^2+^

Panc01 and Panc02 cells were seeded at a density of 1 × 10^5^ cells per well in 6‐well plates and incubated overnight. After incubation, the cells were treated with PBS, LY, CC, CC‐LY, or RPm@CC‐LY for 12 h. To visualize cytoplasmic Ca^2+^ distribution, the cells were washed twice with PBS, stained with Fluo‐4 AM (2 µM) for 30 min, followed by staining with Hoechst 33342 for 15 min. Imaging was performed using an Olympus IX83 Live Microscope (Olympus Life Science, Japan). Intracellular calcium deposits were detected by alizarin red staining. Tumor cells were cultured overnight and then incubated with 2 mL of fresh RPm@CC‐LY solution for 3, 6, 12, or 24 h. After incubation, cells were washed, fixed overnight with 95% ethanol, and stained with 2 mL of alizarin red solution (0.2% in Tris buffer, pH 8.3) for 30 min. The stained cells were observed under an Olympus IX83 Live Microscope (Olympus Life Science, Japan).

### 4.9 Cellular Uptake

Panc01 and Panc02 cells were seeded at a density of 5 × 10^3^ cells per well in a 24‐well plate and cultured overnight. Cells were then incubated with complete medium supplemented with PBS, NR, CC‐LY/NR, or RPm@CC‐LY/NR for 6 h. Following incubation, cells were stained with DAPI for 5 min. Imaging was performed using an Olympus IX83 Live Microscope (Olympus Life Science, Japan), and quantitative uptake analysis was conducted by flow cytometry (CytoFLEX, Beckman, USA).

### 4.10 In Vivo Fluorescence Imaging

To evaluate the active targeting capability of RPm@CC‐LY, Panc02 tumor cells (1 × 10^6^ cells/mouse) were subcutaneously injected into the right thigh of female C57BL/6 mice. Subsequently, ICG, CC‐LY/ICG, or RPm@CC‐LY/ICG (6 mg/kg) was administered via tail vein injection. At various time points (6, 12, 24, and 48 h), the mice were anesthetized and imaged using an IVIS imaging system.

### 4.11 Evaluation of In Vivo Antitumor Activity

Female C57BL/6 mice were subcutaneously inoculated with 0.1 mL of Panc02 cell suspension (5.0 × 10^6^ cells) in the right hind leg to establish a tumor model. When tumor volumes reached 50–80 mm^3^, the mice were randomly divided into six groups: NS, RPm@CC, LY+αPD1, CC‐LY, RPm@CC‐LY, and RPm@CC‐LY+αPD1. Each nanoparticle formulation was administered intravenously at a dose of 1.5 mg/kg LY every other day for one week. Tumor size and body weight were measured every two days. Tumor volumes (mm^3^) were calculated according to the formula: V = (length) × (width)^2^ × 1/2. After two weeks of treatment, major organs (heart, liver, spleen, lung, and kidney) and tumors were harvested for H&E staining, Masson's trichrome staining, TUNEL staining, α‐SMA staining, DLAT staining, p‐Smad3 staining, Fibronectin staining, FAP staining, collagen I and CD3^+^ immunofluorescence staining, CD8^+^ immunofluorescence staining, and immunohistochemical analysis of Ki67. In addition, blood was collected from the mice on day 15 for blood biochemistry and routine hematological analysis.

### 4.12 Statistical Analysis

All results were obtained through independent experiments repeated at least three times. No sample data were excluded. Statistical analysis was performed using GraphPad Prism 10.0 (GraphPad Software Inc., CA, USA). Results are presented as means ± SD. Statistical significance was determined using the Student's t‐test for two‐group comparisons and one‐way ANOVA for multiple‐group comparisons, followed by Tukey's two‐tailed post‐hoc test for pairwise analysis; all tests were two‐sided, and mouse survival curves were analyzed using the Log‐rank (Mantel‐Cox) test. n.s. indicates no statistical significance; ^*^ *p* < 0.05, ^**^ *p* < 0.01, ^***^ *p* < 0.001, ^****^ *p* < 0.0001.

## Author Contributions

J.M.L. and Q.L.W. performed the experiments and analysed the results. J.M.L., Q.L.W., J.D., L.Y., F.X., and Z.G.W. assisted with the figure production and experiment design. J.M.L., Q.L.W., and P.X.C. wrote and edited the manuscript. P.X.C. designed the experiments and supervised the whole project. All authors discussed the results and commented on the manuscript.

## Funding

The work was supported by the Noncommunicable Chronic Diseases‐National Science and Technology Major Project (2023ZD0503000 and 2023ZD0503004), The Regional Innovation and Development Joint Fund Key Project of the National Natural Science Foundation of China (U24A20735), the National Natural Sciences Foundation of China (82473434), the Sichuan Science and Technology Program (2025YFHZ0087), the Science and Technology Project of Sichuan Provincial Health Commission (Clinical Research Special Project JH2023082), the International Science and Technology Cooperation Program of Chengdu Science and Technology Bureau (2024‐YF06‐00011‐HZ and 2022‐GH03‐00004‐HZ), the Health Research Project of Chengdu Eastern New Area Management Committee (202304), the 1.3.5 project for disciplines of excellence from West China Hospital of Sichuan University (ZYYC23006), the Clinical Research Incubation Project of West China Hospital (23HXFH001), the Fundamental and Interdisciplinary Disciplines Breakthrough Plan of the Ministry of Education of China (JYB2025XDXM607), the Tianfu Jincheng Laboratory (Future Medical City, No. TFJC‐2023‐JB004), the Institutional Joint Innovation Fund from Sichuan University and Nuclear Power Institute of China (HG2024220), the Clinical Research Youth Fund of West China Hospital (2025HXFH028), the West China Hospital “Qimingxing” Research Fund for Young Talents (HXQMX0184), the Yunnan Province Key Laboratory of Precision Diagnosis and Treatment for Thoracic Diseases (202449CE340026), the National Key R&D·Program of China (2023YFC3403200 and 2023YFC3403204), and the Ministry of Education University‐Industry Collaborative Education Program (230720523707281). The funders had no role in study design, data collection and analysis, decision to publish, or preparation of the manuscript.

## Conflicts of Interest

The authors declare no conflicts of interest.

## Supporting information




**Supporting File**: advs78436‐sup‐0001‐SuppMat.docx.

## Data Availability

The data that support the findings of this study are available from the corresponding author upon reasonable request.
