# Supplementary material for: Biomimetic Nanoplatform for Fibrosis Remodeling and Anti‐PD‐1 Potentiation via Cu+/Ca2+ Ion Dysregulation and TGF‐β Inhibition in PDAC
Source: Adv Sci (Weinh). 2026 Oct 22:e78436. Online ahead of print. doi: 10.1002/advs.78436 (PMC13690972; doi:10.1002/advs.78436)
Supplement: Supplementary file 1 [file ADVS-9999-e78436-s001.docx]

Supporting Information

**Biomimetic Nanoplatform for Fibrosis Remodeling and Anti-PD-1 Potentiation via Cu^+^/Ca^2+^ Ion Dysregulation and TGF-β Inhibition in PDAC**

*Jianmei Li,^1^ Qinlong Wen,^1,^* Jie Dai, Li Yang, Feng Xiong, Zhigong Wei, Xingchen Peng**

**Figure S1.** Average particle size (n = 3) of hollow mesoporous calcium-doped copper sulfide (CC).

**Figure S2.** Average particle size (n = 3) of CC-LY.

**Figure S3.** X-ray photoelectron spectroscopy (XPS) spectrum of the CC nanoparticles.

**Figure S4.** The sodium dodecyl sulfatepolyacrylamide gel electrophoresis (SDS-PAGE) protein analysis of RAW264.7, Panc01, CC-LY, and a multifunctional biomimetic nanoplatform (RPm@CC-LY) by Coomassie blue staining.

**Figure S5.** Average particle size (n = 3) of RPm@CC-LY.

**Figure S6.** Zeta potentials of CC,CC-LY and RPm@CC-LY.

**Figure S7.** Concentration-dependent standard curve for LY2157299(LY) absorption.

**Figure S8.** Pore size distribution of CC.

**Figure S9.** Pore size distribution of RPm@CC-LY.

**Figure S10.** In vitro release profiles of LY and CC-LY in PBS at pH 7.4 (n = 3).

**Figure S11.** The generation of •OH detected by methylene blue (MB).

**Figure S12.** The cell viability of AML12 cells was analyzed after incubation with varying concentrations of CC for 24 hours (n = 3). Statistical signiﬁcance was determined using the Student’s t-test for two-group comparisons. Significance thresholds: *p < 0.05, **p < 0.01, ***p < 0.001, ****p < 0.0001.

**Figure S13.** The cell viability of AML12 cells was analyzed after incubation with varying concentrations of LY, CC-LY and RPm@CC-LY for 24 hours (n = 3). Statistical signiﬁcance was determined using the Student’s t-test for two-group comparisons. Significance thresholds: *p < 0.05, **p < 0.01, ***p < 0.001, ****p < 0.0001.

**Figure S14.** Flow cytometric analysis images of the Annexin V-FITC/PI-stained AML12 cells treated with different treatments.

**Figure S15.** Apoptosis rate in different groups (n=3). Statistical signiﬁcance was determined using the one-way ANOVA for multiple group comparisons, followed by Tukey’s two-tailed post-hoc test for pairwise analysis, all tests were two-sided. Significance thresholds: *p < 0.05, **p < 0.01, ***p < 0.001, ****p < 0.0001.

**Figure** **S16.** The cell viability of Panc02 cells was analyzed after incubation with varying concentrations of CC for 24 hours (n = 3). Statistical signiﬁcance was determined using the Student’s t-test for two-group comparisons. Significance thresholds: *p < 0.05, **p < 0.01, ***p < 0.001, ****p < 0.0001.

**Figure S17.** The cell viability of Panc02 cells was analyzed after incubation with varying concentrations of LY、CC-LY and RPm@CC-LY for 24 hours (n = 3). Statistical signiﬁcance was determined using the Student’s t-test for two-group comparisons. Significance thresholds: *p < 0.05, **p < 0.01, ***p < 0.001, ****p < 0.0001.

**Figure S18.** The cell viability of Panc01 cells was analyzed after incubation with varying concentrations of CC for 24 hours (n = 3). Statistical signiﬁcance was determined using the Student’s t-test for two-group comparisons. Significance thresholds: *p < 0.05, **p < 0.01, ***p < 0.001, ****p < 0.0001.

**Figure S19.** The cell viability of Panc01 cells was analyzed after incubation with varying concentrations of LY、CC-LY and RPm@CC-LY for 24 hours (n = 3). Statistical signiﬁcance was determined using the Student’s t-test for two-group comparisons. Significance thresholds: *p < 0.05, **p < 0.01, ***p < 0.001, ****p < 0.0001.

**Figure S20.** Flow cytometric analysis images of the Annexin V-FITC/PI-stained Panc01 cells treated with different treatments.

**Figure S21.** Apoptosis rate in different groups (n=3). Statistical signiﬁcance was determined using the one-way ANOVA for multiple group comparisons, followed by Tukey’s two-tailed post-hoc test for pairwise analysis, all tests were two-sided. Significance thresholds: *p < 0.05, **p < 0.01, ***p < 0.001, ****p < 0.0001.

**Figure S22.** Fluorescence images of the live and dead Panc01 cells with various treatments.

**Figure S23.** Panc02 Cell Scratch Assay Photographs Taken at 0, 12, and 24 hours.

**Figure S24.** Healing rate of different groups (n=3). Statistical signiﬁcance was determined using the Student’s t-test for two-group comparisons. Significance thresholds: *p < 0.05, **p < 0.01, ***p < 0.001, ****p < 0.0001.

**Figure S25.** Panc01 Cell Scratch Assay Photographs Taken at 0, 12, and 24 hours.

**Figure S26.** Healing rate of different groups (n=3). Statistical signiﬁcance was determined using the Student’s t-test for two-group comparisons. Significance thresholds: *p < 0.05, **p < 0.01, ***p < 0.001, ****p < 0.0001.

**Figure S27.** Fluorescence images of different drugs taken up by Panc01 cells.

**Figure S28.** Flow cytometry analysis of different drugs uptake by Panc01 cells.

**Figure S29.** Fluorescence images of DCFH-DA-stained Panc01 cells after treatment with various drugs for 24 h.

**Figure S30.** Fluorescence images of the activity in Panc01 cells of mitochondria under various treatments. Scale bar = 20 μm.

**Figure S31.** Fluorescence images of the expression levels of γ-H2AX in Panc01 cells with various treatments.

**Figure S32.** Intracellular Ca^2+^ ions accumulation in Panc01 cells.

**Figure S33.** Panc01 cells were incubated with RPm@CC-LY for varying periods and then stained with alizarin red to identify in vitro cellular products that exhibited red areas of calcification.

**Figure S34.** Western-blotting analysis and semi-quantitative results of DLAT, FDX1, and LIAS.

**Figure S35.** Tumor growth curves of the mice bearing Panc02 tumors in different groups (n =6). Statistical signiﬁcance was determined using the one-way ANOVA for multiple group comparisons, followed by Tukey’s two-tailed post-hoc test for pairwise analysis, all tests were two-sided. Significance thresholds: *p < 0.05, **p < 0.01, ***p < 0.001, ****p < 0.0001.

**Figure S36.** Tumor growth curves of each mouse bearing Panc02 tumors in LY group (n = 6).

**Figure S37.** Tumor growth curves of each mouse bearing Panc02 tumors in αPD1 group (n = 6).

**Figure S38.** Tumor growth curves of each mouse bearing Panc02 tumors in RPm@CC group (n = 6).

**Figure S39.** Tumor growth curves of each mouse bearing Panc02 tumors in LY+αPD1 group (n = 6).

**Figure S40.** Representative photographs of isolated tumors (n = 5). I: Control; II: RPm@CC; III: LY+αPD1; IV:CC-LY; V: RPm@CC-LY; VI: RPm@CC-LY+αPD1.

**Figure S41.** Representative photographs of isolated tumors (n = 5). VII:LY; VIII:αPD1.

**Figure S42.** Weight of tumors in different treatment groups (n = 5 ). Statistical signiﬁcance was determined using the Student’s t-test for two-group comparisons. Significance thresholds: *p < 0.05, **p < 0.01, ***p < 0.001, ****p < 0.0001.

**Figure S43.** Body weight fluctuations during treatment (n = 6). Statistical signiﬁcance was determined using the Student’s t-test for two-group comparisons. Significance thresholds: *p < 0.05, **p < 0.01, ***p < 0.001, ****p < 0.0001.

**Figure S44.** Survival patterns of each group with different treatments (n = 6).

**Figure S45.** H&E staining of tumors after different treatments, and immunofluorescence images of α-SMA, TUNEL and DLAT in tumor sections. (n = 3). Scale bar, 50 μm.

**Figure S46. a**) Immunohistochemical staining of pSmad3 in the excised tumors. Scale bar = 50μm. **b**) Quantitative analysis of positive ratios of pSmad3 staining (n=3). Statistical signiﬁcance was determined using the Student’s t-test for two-group comparisons. Significance thresholds: *p < 0.05, **p < 0.01, ***p < 0.001, ****p < 0.0001.

**Figure S47.** **a**) Immunohistochemical staining of FAP in the excised tumors. Scale bar = 50 μm. **b**) Quantitative analysis of positive ratios of FAP staining (n=3). Statistical signiﬁcance was determined using the Student’s t-test for two-group comparisons. Significance thresholds: *p < 0.05, **p < 0.01, ***p < 0.001, ****p < 0.0001.

**Figure S48. a**) Immunohistochemical staining of the intratumoral fibronectin expression. Scale bar = 50 μm. **b**) Quantitative analysis of positive ratios of fibronectin staining (n=3). Statistical signiﬁcance was determined using the Student’s t-test for two-group comparisons. Significance thresholds: *p < 0.05, **p < 0.01, ***p < 0.001, ****p < 0.0001.

**Figure S49.** Immunohistochemical staining of Masson in the excised tumors. Scale bar = 50 μm.

**Figure S50.** Immunohistochemical staining of collagen I and CD3^+^ T lymphocytes in the excised tumors. Scale bar = 50 μm.

**Figure S51.** Immunohistochemical staining of Ki67. Scale bar = 50μm.

**Figure S52.** In vitro analysis of hemolysis. **a** microscopic photograph showing representative erythrocytes cultures with various drugs. I:normal saline (NS, negative control);II: distilled water (DW, positive control); III:LY; IV:CC; V:CC-LY; VI:RPm@CC-LY. **b** Photographs demonstrating hemolysis. **c** UV-Vis absorption spectra for each group. **d** the hemolysis rate for each group.

**Figure S53.** Representative H&E-stained images from major organ tissue section (scale bar = 50 μm).

**Figure S54.** The systemic toxicity of various treatments was analyzed in vivo by examining biochemical markers and routine blood indicators.

**Figure S55.** Immunofluorescence images of CD8^+^T in tumor sections. scale bar = 50 μm.

**Figure S56.** Quantitative analysis of Mature DCs (CD80^+^CD86^+^) s in the spleen (n=3). Statistical signiﬁcance was determined using the Student’s t-test for two-group comparisons. Significance thresholds: *p < 0.05, **p < 0.01, ***p < 0.001, ****p < 0.0001.

**Figure S57.** Flow cytometric of Mature DCs (CD80^+^CD86^+^) s in the spleen.

**Figure S58.** Flow cytometric of Mature DCs (CD80^+^CD86^+^) in tumor.

.

**Figure S59.** Quantitative analysis of Mature DCs (CD80^+^CD86^+^) in tumor (n=3). Statistical signiﬁcance was determined using the Student’s t-test for two-group comparisons. Significance thresholds: *p < 0.05, **p < 0.01, ***p < 0.001, ****p < 0.0001.

**Figure S60.** Flow cytometric of Mature DCs (CD80^+^CD86^+^) in lymph nodes.

**Figure S61.** Quantitative analysis of Mature DCs (CD80^+^CD86^+^) in lymph nodes (n=3). Statistical signiﬁcance was determined using the one-way ANOVA for multiple group comparisons, followed by Tukey’s two-tailed post-hoc test for pairwise analysis, all tests were two-sided. Significance thresholds: *p < 0.05, **p < 0.01, ***p < 0.001, ****p < 0.0001.

**Figure S62.** Flow cytometric of CD8^+^ T cells (CD3^+^CD8^+^) in tumor samples obtained from mice.

**Figure S63.** Quantitative analysis of CD8^+^ T cells (CD3^+^CD8^+^) in tumor samples obtained from mice (n=3). Statistical signiﬁcance was determined using the Student’s t-test for two-group comparisons. Significance thresholds: *p < 0.05, **p < 0.01, ***p < 0.001, ****p < 0.0001.

**Figure S64.** Flow cytometric of CD8^+^ T cells in the spleen.

**Figure S65.** Quantitative analysis of CD8^+^ T cells in the spleen (n=3). Statistical signiﬁcance was determined using the Student’s t-test for two-group comparisons. Significance thresholds: *p < 0.05, **p < 0.01, ***p < 0.001, ****p < 0.0001.

**Figure S66.** Flow cytometric of CD80^+^CD206^+^ in tumor tissues.

**Figure S67.** Quantitative analysis of M1 in tumor tissues (n=3). Statistical signiﬁcance was determined using the Student’s t-test for two-group comparisons. Significance thresholds: *p < 0.05, **p < 0.01, ***p < 0.001, ****p < 0.0001.

**Figure S68.** Quantitative analysis of M2 in tumor tissues (n=3). Statistical signiﬁcance was determined using the Student’s t-test for two-group comparisons. Significance thresholds: *p < 0.05, **p < 0.01, ***p < 0.001, ****p < 0.0001.

**Figure S69.** Flow cytometric of CD80^+^CD206^+^ in the spleen.

**Figure S70.** Quantitative analysis of M1 in the spleen (n=3). Statistical signiﬁcance was determined using the one-way ANOVA for multiple group comparisons, followed by Tukey’s two-tailed post-hoc test for pairwise analysis, all tests were two-sided. Significance thresholds: *p < 0.05, **p < 0.01, ***p < 0.001, ****p < 0.0001.

**Figure S71.** Quantitative analysis of M2 in the spleen (n=3). Statistical signiﬁcance was determined using the one-way ANOVA for multiple group comparisons, followed by Tukey’s two-tailed post-hoc test for pairwise analysis, all tests were two-sided. Significance thresholds: *p < 0.05, **p < 0.01, ***p < 0.001, ****p < 0.0001.

**Figure S72.** Levels of IFN-γ in serum from each group of mice after treatment (n=3). Statistical signiﬁcance was determined using the Student’s t-test for two-group comparisons. Significance thresholds: *p < 0.05, **p < 0.01, ***p < 0.001, ****p < 0.0001.

**Figure S73.** Levels of IL-6 in serum from each group of mice after treatment (n=3). Statistical signiﬁcance was determined using the Student’s t-test for two-group comparisons. Significance thresholds: *p < 0.05, **p < 0.01, ***p < 0.001, ****p < 0.0001.

**Figure S74.** Levels of TNF-α in serum from each group of mice after treatment (n=3). Statistical signiﬁcance was determined using the Student’s t-test for two-group comparisons. Significance thresholds: *p < 0.05, **p < 0.01, ***p < 0.001, ****p < 0.0001.

**Figure** **S75.** Body weight fluctuations during treatment (n = 6). Statistical signiﬁcance was determined using the Student’s t-test for two-group comparisons. Significance thresholds: *p < 0.05, **p < 0.01, ***p < 0.001, ****p < 0.0001.

**Figure S76.** Representative photograph of primary tumors in each group after treatments (n = 5). I: Control; II: LY+αPD1; III: RPm@CC-LY; IV: RPm@CC-LY+αPD1.

**Figure S77.** Representative photograph of re-challenge tumors in each group after treatments (n = 5). I: Control; II: LY+αPD1; III: RPm@CC-LY; IV: RPm@CC-LY+αPD1.

**Figure S78.** Representative flow cytometric analysis of effector memory T cell (Tem, CD44^+^ CD62L^−^) from CD8^+^ T cells in re-challenge tumors after different treatments.

**Figure S79.** Representative quantification of effector memory T cell (Tem, CD44^+^ CD62L^−^) from CD8^+^ T cells in re-challenge tumors after different treatments (n=3). Statistical signiﬁcance was determined using the one-way ANOVA for multiple group comparisons, followed by Tukey’s two-tailed post-hoc test for pairwise analysis, all tests were two-sided. Significance thresholds: *p < 0.05, **p < 0.01, ***p < 0.001, ****p < 0.0001.

**Figure S80.** Flow cytometric of NK cells in re-challenge tumor.

**Figure S81.** Quantitative analysis of NK cells in re-challenge tumor (n=3). Statistical signiﬁcance was determined using the one-way ANOVA for multiple group comparisons, followed by Tukey’s two-tailed post-hoc test for pairwise analysis, all tests were two-sided. Significance thresholds: *p < 0.05, **p < 0.01, ***p < 0.001, ****p < 0.0001.

**Figure S82.** Flow cytometric of NK in the spleen.

**Figure S83.** Quantitative analysis of NK cells in the spleen (n=3). Statistical signiﬁcance was determined using the one-way ANOVA for multiple group comparisons, followed by Tukey’s two-tailed post-hoc test for pairwise analysis, all tests were two-sided. Significance thresholds: *p < 0.05, **p < 0.01, ***p < 0.001, ****p < 0.0001.

**Figure S84. a**) Additional immunohistochemical staining demonstrating FAP expression within re-challenge tumors. Scale bar = 50 μm. **b**) Quantitative analysis of positive ratios of FAP staining (n=3). Statistical signiﬁcance was determined using the Student’s t-test for two-group comparisons. Significance thresholds: *p < 0.05, **p < 0.01, ***p < 0.001, ****p < 0.0001.

**Figure S85. a**) Additional immunohistochemical staining demonstrating fibronectin expression within re-challenge tumors. Scale bar = 50 μm. **b**) Quantitative analysis of positive ratios of fibronectin staining (n=3). Statistical signiﬁcance was determined using the Student’s t-test for two-group comparisons. Significance thresholds: *p < 0.05, **p < 0.01, ***p < 0.001, ****p < 0.0001.

**Experimental Sections**

**1. Cell Culture and animals:** Tumor cell lines, including Panc01 (human pancreatic cancer), Panc02 (mouse pancreatic cancer), AML12 (mouse hepatocyte) were obtained from the Cell Bank of the Chinese Academy of Sciences (Shanghai, China). Panc01 and Panc02 cells were cultured in DMEM (Gibco) supplemented with 1% penicillin (Boster), 1% streptomycin (Boster), and 10% fetal bovine serum (FBS, Viva Cell) at 37°C in a humidified atmosphere containing 5% CO_2_. For subsequent experiments, cells were immediately transferred after seeding to an anaerobic incubator (Thermo Scientific, USA) containing 94.5% N_2_, 5% CO_2_, and 0.5% O_2_ to induce hypoxia.

**2. Animal models:** Female C57 mice aged 6–8 weeks were purchased from Chengdu Huafukang and Yaokang Biotechnology Co., Ltd. and housed in a specific pathogen-free facility. All mice were maintained under controlled conditions with a 12-hour light/dark cycle, a constant temperature (18°C–22°C), and humidity (50%–60%), with ad libitum access to standard chow and water. All animal procedures were approved by the Animal Care and Use Committee of West China Hospital, Sichuan University (Approval No.: 20250217007) and conducted in compliance with the ARRIVE guidelines.

**3. Synthesis of CC-LY:** First dissolve LY2157299 (2 mg) in 1 mL of deionized water. Add this solution dropwise to 4 mL of CC (2 mg mL^-1^) under ultrasonic treatment. After magnetic stirring for 12 hours, collect the CC-LY by centrifuging at 10,000 rpm for 20 minutes. Wash the collected sample three times with deionized water, and then freeze-dry it.

**4. Preparation of Cancer and Macrophage Cell Membrane:** Lyse the cells in a cell membrane protein extraction solution at 4 °C for 15 minutes. Perform five cycles of rapid freezing in liquid nitrogen followed by thawing at 37 °C. After centrifugation at 1,000 g, collect the supernatant. Finally, isolate the cell membranes by centrifugation at 14,000 g.

**5. LY Release Assay:** Evaluate the in vitro release of LY using a dialysis method. Briefly, dissolve samples containing equal amounts of LY in PBS (pH 5.0, 6.5, or 7.4) and place them in dialysis bags (molecular weight cutoff: 3,500 Da). Incubate the bags in an oscillating water bath at 100 rpm. At predetermined time points, withdraw 2 mL of release medium and replace it with an equal volume of fresh medium. Measure the absorbance of each sample at 239 nm using a UV spectrophotometer (UV-5800 PC, Shanghai Metasys Instruments Co., Ltd., Shanghai, China). Calculate and plot the cumulative drug release rate.

**6. In Vitro Cytotoxicity Assay:** The cytotoxicity of nanoparticles was evaluated using the MTT assay. AML12, Panc01, and Panc02 cells were seeded in 96-well plates (1 × 10^4^ cells/well) and incubated for 12 hours. The cells were then treated with varying concentrations of LY, CC, CC-LY, or RPm@CC-LY for 24 hours. Cell viability was assessed by measuring absorbance at 490 nm using a microplate reader (FLUOstar Omega).

**7. Apoptosis assays:** Panc01, Panc02, and AML12 cells were cultured overnight in six-well plates and treated with PBS, LY, CC, CC-LY, or RPm@CC-LY for 24 hours. Apoptosis was detected using the Annexin V-FITC/PI apoptosis assay kit (4A BIOTECH, China) and quantified by flow cytometry (CytoFLEX, Beckman, USA).

**8. Live/dead assay:** Panc01 and Panc02 cells were cultured overnight in 6-well plates and treated with PBS, LY, CC, CC-LY, or RPm@CC-LY for 24 hours. Cells were then stained using the Calcein/PI Live/Dead Viability Assay Kit (Beyotime, China), and images were captured with an Olympus IX83 Live Microscope (Olympus Life Science, Japan).

**9. γ-H2AX immunofluorescence assay:** Panc01 and Panc02 cells were seeded overnight in 6-well plates and treated with PBS, LY, CC, CC-LY, or RPm@CC-LY for 4 hours. Immunofluorescence staining for γ-H2AX was conducted using the γ-H2AX Immunofluorescence DNA Damage Detection Kit (Beyotime, China). Images were acquired using an Olympus IX83 Live Microscope (Olympus Life Science, Japan).

**10. In Vitro Migration Assay:** The in vitro migration capacity of pancreatic cancer cells was evaluated using a scratch assay. Cells were seeded at a density of 5 × 10^5^ cells per well in 6-well plates and cultured until 90% confluence. A linear scratch (“wound”) was then created in the monolayer using the tip of a sterile pipette. Detached cells were removed by washing, and fresh medium containing various NPs at an LY concentration of 10μg mL^-1^ was added. Images of the wound area were captured at 0, 12, and 24 hours using an Olympus IX83 Live Microscope (Olympus Life Science, Japan), and migration rates were calculated based on wound closure.

**11. In Vitro Hemolysis Assay:** To assess hemolysis, 1 mL of a 2% (v/v) red blood cell suspension was mixed with 1 mL of either NS, LY, CC, CC-LY, or RPm@CC-LY, and incubated at 37°C for 4 hours. Cells incubated in double-distilled water and NS served as positive and negative controls, respectively. After incubation, samples were centrifuged at 3000 rpm for 6 minutes, and the optical density (OD) of each supernatant was measured at 540 nm using a UV-5800 PC spectrophotometer. The hemolysis rate was calculated using the following formula:

Hemolysis rate (%) = (OD value of the experimental group − OD value of the saline group)/ (OD value of the positive control group − OD value of the saline group) × 100%

**12. Flow cytometry analysis:** Single-cell suspensions were prepared by digesting isolated tumor tissue or spleen. The immune cells were construed following the standard protocol. To prevent non-specific binding, the cell suspension was incubated with BD Fc Block^TM^ purified anti-mouse CD16/CD32 monoclonal antibody (BD Pharmingen^TM^, Catalog: 553141) for 15 min at 4 °C. Then, cells were stained with surface antibodies: CD45-APC-CY7 (BD Pharmingen^TM^, Catalog: 557659), CD3-BUV395 (BD Pharmingen^TM,^ Catalog: 563565), CD4-BUV661 (BD Pharmingen^TM^, Catalog: 741461), CD8-BV711 (BD Pharmingen^TM^, Catalog: 563046), NK1.1-BV750(BD Pharmingen^TM^, Catalog: 746876), CD62L-FITC (BD Pharmingen^TM^, Catalog: 561917), CD44-PerCP-Cy5.5 (BD Pharmingen^TM^, Catalog:560570), CD11b-BV421 (BD Pharmingen^TM^, Catalog: 562605), F4/80-BV605 (BD Pharmingen^TM^, Catalog: 743281), D86-BUV737 (BD Pharmingen^TM^, Catalog: 741737) , CD11c-BUV563 (BD Pharmingen^TM^, Catalog: 749091), MHC II-R718 (BD Pharmingen^TM^, Catalog: 752163), CD80-BV510 (BD Pharmingen^TM,^ Catalog: 740130). Following fixation and permeabilization, cells were stained with intracellular markers: CD206-AF647 (BD Pharmingen^TM^, Catalog: 566250). Finally, using BD FACSymphony^TM^ A5 for filtering and detection of stained cells and FlowJo 10.4 for data analysis. Flow cytometry gating strategy involved selecting single cells based on FSC-A/SSC-A, removing dead cells using a viability dye, and identifying specific cell populations through marker expression (e.g., CD45^+^ for immune cells).

**13. Serum Collection and Enzyme-Linked Immunosorbent Assay (ELISA):** For each group, blood samples were collected via ocular phlebotomy into dry EP tubes on day 14 after treatment. The samples were centrifuged at 4 °C following standard procedures, and the upper serum was carefully collected. The levels of cytokines, including IFN-γ, IL-6, and TNF-α, in the serum were measured using a commercial ELISA kit (ZCIBIO Technology, Shanghai, China).

**14. In Vivo Assessment of Sustained Antitumor Immunity in re-challenge Tumor Models:** Female C57BL/6 mice bearing Panc02 tumors (right thigh, 40 mm^3^) were randomly divided into four groups: saline, LY+αPD1, RPm@CC-LY, and RPm@CC-LY+αPD1. Each nanoparticle formulation was administered intravenously at a dose equivalent to 1.5 mg kg^-1^ LY every other day for one week. On day 0, Panc02 cells (5×10^5^ cells/mouse) were subcutaneously inoculated into the left leg. Tumor volumes were monitored regularly. After treatment, tumor tissues were harvested for histopathological analysis, including H&E staining, TUNEL staining, α-SMA staining, Fibronectin staining, FAP staining, DLAT staining, CD8^+^ immunofluorescence staining, and Ki67 immunohistochemistry. For immune evaluation, cell suspensions were incubated with BD Fc Block™ purified anti-mouse CD16/CD32 monoclonal antibody (BD Pharmingen™, Cat. No.: 553141) at 4°C for 15 minutes. Subsequently, cells were stained with surface antibodies: CD45-APC-CY7 (BD Pharmingen™, Cat. No.: 557659), CD3-BUV395 (BD Pharmingen™, Cat. No.: 563565), CD4-BUV661 (BD Pharmingen™, Cat. No.: 741461), CD8-BV711 (BD Pharmingen™, Catalog No.: 563046), NK1.1-BV750 (BD Pharmingen™, Catalog No.: 746876), CD62L-FITC (BD Pharmingen™, Catalog No.: 561917), CD44-PerCP-Cy5.5 (BD Pharmingen™, Catalog No.: 560570). Stained cells were filtered and detected using the BD FACSymphony™ A5 flow cytometer, with data analyzed via FlowJo 10.4 software. The flow cytometry gating strategy involved selecting single cells via FSC-A/SSC-A parameters, excluding dead cells using a viability dye, and identifying specific cell populations based on marker expression (e.g., CD45^+^ for immune cells). Serum samples were also collected and analyzed with ELISA kits to quantify cytokines, including TNF-α, IL-6, and IFN-γ.
